# Cellular landscape of avian intestinal organoids revealed by single cell transcriptomics

**DOI:** 10.1038/s41598-025-95721-4

**Published:** 2025-04-02

**Authors:** Jianxuan Sun, Dominika Borowska, James J. Furniss, Kate Sutton, Daniel J. Macqueen, Lonneke Vervelde

**Affiliations:** 1 The Roslin Institute and Royal (Dick) School of Veterinary Studies, The University of Edinburgh, Midlothian, UK; 2 Royal GD, Deventer, The Netherlands

**Keywords:** Gastrointestinal models, Transcriptomics

## Abstract

Studies of the avian gastrointestinal tract, where nutrient absorption and key host-pathogen interactions occur, have been strongly enabled by the development of intestinal organoid models. Here we report a single cell transcriptomic atlas of intestinal organoid cells derived from embryos of broiler and layer chickens, capturing mesenchymal, epithelial, endothelial, immune and neuronal cell lineages. Eight inferred mesenchymal subpopulations reflect anatomically distinct intestinal layers, including fibroblasts, telocytes, myofibroblasts, smooth myocytes, pericytes, and interstitial cells of Cajal. Identified heterogeneity within the epithelial lineage included enterocytes, goblet cells, Paneth cells, tuft cells, and diverse enteroendocrine cell subtypes. Additionally, we identified candidate macrophages, monocytes, γδ T cells, NK cells and granulocytes. Layer and broiler organoids showed significant differences in cell-specific transcriptome, most pronounced in epithelial cells, pointing to divergent selection on intestinal physiology. Our analysis finally provides a catalogue of novel cell marker genes to enable future research of chicken intestinal organoids.

**Supplementary Information:**

The online version contains supplementary material available at 10.1038/s41598-025-95721-4.

## Introduction

As the key entry point for nutrients and pathogens^1^, understanding and maintaining the gastrointestinal tract is crucial for the poultry industry. A lack of representative cell culture lines has historically hindered in vitro studies of the avian gut, including analyses of host-pathogen interactions^2^. The development of three-dimensional (3D) mini-gut models has revolutionized the study of enteric diseases and intestinal health. These models can be derived from intestinal crypts, forming enteroids, which consist exclusively of epithelial cells. Alternatively, intestinal organoids are derived from induced pluripotent stem cells (iPSCs) and contain both epithelial and mesenchymal lineages, offering a more complex system. Both enteroids and organoids have a central lumen surrounded by a single layer of highly polarized epithelial cells, with their basolateral side in contact with the extracellular matrix scaffold. A landmark study in 2009 reported the generation of 3D intestinal enteroids derived from *LGR5*^+^ intestinal stem cells in mice^3^. The first chicken 3D enteroid model was generated in a similar fashion as mice, grown in Matrigel and media composition that included exogenous growth factors. However, these models lack the characteristic multi-lobulated structure observed in mammalian enteroids^4^. A more comprehensive chicken mini-gut model with multiple villus-crypt structures was established in 2021. This model differs significantly from the mammalian counterparts. Notably, the chicken mini-gut model was generated from the villi of embryonic day (ED) 18–19 chicks in contrast to mammalian crypt-derived enteroids^3^. Studies have shown that intestinal crypts are at a rudimentary stage at ED18-19 and in-situ hybridization has revealed the presence of *LGR5*-expressing stem cells distributed throughout both the villi and crypts in the small intestine^5^. Hence, by isolating the villi, the chicken mini-gut model consists of stem cells while retaining the underlying lamina propria cells. In addition, the model does not require the presence of an extracellular matrix or addition of exogenous growth factors, such as Noggin and R-spondin^6^. Instead, the released villi seal up to form a 3D structure that floats in basic culture media, retaining a apical-out orientation containing multiple epithelial cell lineages with barrier function, and the underlying lamina propria consisting of immune cell and mesenchymal cell lineages, which possibly produce the growth factors to sustain epithelial cell proliferation and maintenance^7^. Thus, the chicken mini-gut model offers a more complex and physiologically relevant system and hence are defined as organoids. Despite showing limited survival time and inability to passage^8^, chicken 3D organoids are the most physiologically-relevant in vitro model available for studying intestinal health, truly resembling the intestinal tissue in vivo^9^.

The cellular composition of chicken 3D intestinal organoids has been characterised using immunostaining and bulk RNA-Seq however, due to a lack of reagents the true complexity of the model in unknown^7^. Recent studies have leveraged single cell RNA-Seq (scRNA-Seq) to report cellular heterogeneity within both human^10^ and mouse intestinal enteroids^6,11^. In this study, we report a comprehensive scRNA-Seq atlas of chicken 3D intestinal organoids derived from broiler and layer embryos. We explore and report the complex cellular dynamics of chicken organoids, identifying mesenchymal, epithelial and leukocyte heterogeneity, emphasizing the complexity of extrapolating research on organoids to in vivo conditions. Comparative analysis of cell-resolved transcriptomes further revealed differences between broiler and layer organoids, pointing towards differential selection impacting the gastrointestinal tract.

## Materials and methods

### Animals

Fertile Hy-Line Brown Layers and Roslin Broiler eggs (*Gallus gallus*) at embryonic day 19 (ED19) were sourced from the National Avian Research Facility, University of Edinburgh, UK. The Roslin broiler is a closed inter-crossed population derived from a commercial Cobb female parent and the commercial Hubbard M99 male broiler.

Ethical approvals were obtained from The Roslin Institute’s and University of Edinburgh’s Animal Welfare Ethics Review Board. The humane culling of embryos was conducted under the authorisation of a UK Home Office Project License (PE263A4FA) and adhered to the guidelines and regulations of the UK Home Office Animals (Scientific Procedures) Act 1986.

### Generation of chicken three-dimensional organoids

Duodenum, jejunum, and ileum were extracted from chicken embryos (ED19) and immediately placed together in phosphate-buffered saline (PBS, Mg^2+^ and Ca^2+^ free) until further processing. For each independent culture, intestines from four embryos were pooled. The organoids were generated following Nash et al.^7^. Briefly, intestinal tissues were longitudinally opened and cut into 3 mm pieces. To release the villi, the tissues underwent enzymatic digestion using collagenase from *Clostridium histolyticum* Type IA (0.2 mg/mL, Merck) for 50 min with shaking at 200 rpm, 37 °C in 50 mL falcon tubes. After digestion, the villi were further released using mechanical dissociation by rapidly flicking the falcon tubes in a downward movement up to six times. The villi were collected by applying the digestion supernatant onto a 70 μm cell strainer (Corning, UK). The strainer was inverted onto a Petri dish and washed 4 times with 20 mL of DMEM (Gibco, UK) to collect the villi. After each collection the digested tissues underwent additional mechanical dissociation to release more villi. The collected villi were pelleted at 107 x g for 4 min. Up to 50 K villi were captured from the four intestines. Half of the collected villi were seeded at 10 K villi in Petri dishes with 12 mL of Floating Organoid Media (FOM), consisting of Advanced DMEM/F12 supplemented with 1X B27 Plus, 10 mM HEPES, 2 mM L-Glutamine, and 50 U/mL Penicillin/Streptomycin (Thermo Fisher Scientific, Paisley, UK), whereas the other half were processed for scRNA-Seq. The organoids were cultured at 37 °C with 5% CO_2_ in Petri dishes to remove fibroblasts. The following day, organoids were collected, washed in Advanced DMEM/F12, re-plated in Petri dishes and cultured for an additional two days at 37 °C with 5% CO_2_. The organoids on day 3 (D3) of culture were dissociated and sorted using the same procedures as the fresh villi, described below.

### Villi and organoid dissociation

The villi (Day 0, D0) were dissociated into single cells for fluorescence-activated cell sorting (FACS). Dissociation was performed using 0.25% Trypsin-EDTA solution (1X, Gibco, UK) for 15 min at 37 °C, with gentle pipetting every 5 min. The reaction was quenched with addition of DMEM containing 10% FBS (Gibco, UK). Single cells from the dissociated villi were washed twice in PBS supplemented with 1% BSA (Sigma-Aldrich, UK) at 400×*g* for 5 min at 4 °C, filtered through a 35 μm cell strainer, and resuspended in collection buffer consisting of PBS with 1% BSA and 0.4 U/µL of Protector RNase inhibitor (3335399001, Roche). At D3 of culture, the organoids were dissociated using the same procedure as described for the villi. Cell viability and counts were assessed using a hemacytometer and Trypan blue exclusion method. The cells were maintained on ice before FACS was performed using a Bigfoot Cell Sorter (Thermo Fisher Scientific, UK). Sytox™ Blue live/dead stain (Thermo Fisher Scientific, UK) was added to the cells 5 min prior to cell collection. Collection gate was applied to isolate single cells and live cells. Cells were collected in low retention 1.5 mL Eppendorf tubes containing 100 µL of collection buffer.

### Library generation and sequencing

scRNA-seq libraries were prepared by the Roslin Institute Genomics Platform Facility, using the 10X Genomics Chromium Next GEM Single Cell 3’ Kit (v3.1) following the manufacturer’s protocol. FACS sorted cells for eight samples (*n* = 2 per broiler and layer at of isolation (D0) and D3) were re-counted using a hemacytometer and diluted to 700-1,200 cells per µL. Each sample was loaded onto a Chromium Chip G lane targeting 5,000 cells per sample. After encapsulation by gel beads in emulsion (GEMs), the mRNA was captured, barcoded and reverse transcribed, before the emulsion was broken and the cDNA pooled, purified and amplified using 12 PCR cycles. The cDNA was fragmented, end-repaired and size-selected to optimise amplicon size, followed by adaptor ligation and sample indexing (11 PCR cycles). The resulting libraries were pooled in equimolar concentrations and sequenced on an Illumina NextSeq 2000 using NextSeq 1000/2000 P3 Reagents (100 cycles) v3 Kit, over two P3 flow cells. PhiX Control v3 library was spiked at a concentration of ~ 1%.

### Mapping and count matrix generation

Illumina scRNA-Seq reads were quality assessed using FastQC v0.11.9^12^ and aligned to the unmasked paternal white leghorn layer genome (GCF_016700215.2; Ensembl Genome Browser release 110 (https://www.ensembl.org/Gallus_gallus_GCA_016700215.2/Info/Index) using StarSolo v2.7.10a^13^. The same tool was used to attribute reads to cellular barcodes and de-duplicate unique molecular identifiers (UMIs). The analysis was restricted to protein coding genes by specifying annotations with a gene_biotype of “protein coding”. The genome index was generated with standard settings and “sjdbOverhang” set to 99 to account for the 100 bp read length. Reads were mapped with the “STAR” command with the following settings: soloCBstart = 1, soloCBlen = 16, soloUMIstart = 17, soloUMIlen = 12, soloBarcodeReadLength = 0, outFilterScoreMinOverLread = 0.66, outFilterMatchNminOverLread = 0.66, soloUMIfiltering = MultiGeneUMI_CR, soloCellFilter = EmptyDrops_CR, limitOutSJcollapsed = 2500000, soloCellReadStats = Standard. Detailed mapping and count statistics are provided in Supplementary Table 1.

### Filtering and quality control

Initial filtering was conducted in StarSolo v2.7.10a to rank UMIs and gene counts per cells. To filter out cellular barcodes associated with low UMI or gene counts, indicative of empty droplets, we employed an EmptyDrop-like method (soloCellFilter = EmptyDrops_CR) with default parameters. Subsequent quality control was carried out per sample using Seurat v4.0.6^14^. Background RNA was removed using SoupX^14^. Next, cells with gene counts < 200 and/or exhibiting > 5% read counts to mitochondrial genes were excluded. The “SCTransform” function was applied to normalize the data. This was followed by centring, scaling, principal component analysis (PCA) and an initial high resolution clustering (20 principal components; resolution of 2.5) using the Seurat “FindClusters” command to identify additional clusters of empty droplets^15^. Populations that lacked distinguishing markers (see section: ‘Differential gene expression tests’) were removed as likely empty droplets or poor-quality cells. Additionally, doublets were identified and removed using the R package ScDblFinder v1.10.0^16^. A manual quality control step was performed to remove further potential doublets once cell identity was established (described in section: ‘Assignment of cellular identity’).

### Assignment of cellular identity

The eight samples were integrated into a single Seurat object using the “merge” function. Harmony v0.1.0^17^ was used to reduce confounding sample-specific batch effects. Default parameters were utilized for clustering (resolution set to 0.8). Clusters identified in the integrated Seurat dataset were assigned to major cell lineages using a priori defined marker genes (Supplementary Table 2). Gene annotations were taken from the Ensembl Genome Browser annotation for paternal white leghorn layer. For genes lacking annotations, the nearest Ensembl annotated broiler chicken (GCA_016699485.1; https://www.ensembl.org/Gallus_gallus/Info/Index) was adopted (Supplementary Table 3). For the additional cell lineage-specific analysis, all major cell populations (i.e. Supplementary Table 4) were split into separate Seurat objects, followed by additional clustering using default parameters. Each subpopulation was then assigned an identity based on canonical marker genes sourced from the literature (Supplementary Table 2). Major cell lineage markers (Supplementary Table 2; see section: ‘Differential gene expression tests’) and detailed statistics including the number and proportion of each cell type per sample are provided in Supplementary Table 5. To explore heterogeneity within the identified enteroendocrine cell lineage, relevant subpopulations were isolated from epithelial cells and underwent processing using the same workflow (marker genes used to assign cell identity provided in Supplementary Table 6).

### Differential gene expression tests

To identify gene expression profiles and marker genes for different cell populations, a differential gene expression (DGE) test was performed using the Seurat “FindAllMarkers” function^18^ based on the Wilcoxon rank sum test. This approach involves comparing the expression of each gene in a specific cell lineage with its expression in all other cells (results in Supplementary Table 4). Genes with Bonferroni-adjusted *p* < 0.05 and average log2FC > 0.50 were considered significantly differentially expressed. Genes significantly upregulated in each cell lineage were considered markers. For the cell lineage-specific DGE analysis, the background was set as all cells analysed for the major cell lineage being examined (resultant marker genes shown in Supplementary Tables 7–11). Summary statistics of the identified cell subpopulations across the eight samples are provided in Supplementary Table 12. For the enteroendocrine specific DGE analysis (results in Supplementary Table 13), the background was set as all enteroendocrine cells (additional summary statistics provided in Supplementary Table 12).

To examine cell-specific gene expression differences between broiler and layer D3 organoids, we performed DGE tests for: (i) the major defined cell lineages (results in Supplementary Table 14) and (ii) the six different epithelial cell subpopulations (results in Supplementary Table 15), contrasting the broiler samples and layer samples using the “FindMarkers” function in Seurat. Genes with FDR-adjusted *p* < 0.05 and average log2FC > 0.50 were considered differentially expressed.

### GO enrichment analyses

Gene Ontology (GO) enrichment tests were performed using the R package clusterProfiler v4.8.3^19^, on differentially expressed genes (DEGs) between epithelial subpopulations of broiler and layer organoids (DEGs listed in Supplementary Table 15). GO analyses were limited to Biological Function GO terms and performed separately for each of six identified epithelial subpopulations, separately for broiler and layer upregulated genes, with the background gene sets representing all genes expressed across all epithelial cells (results in Supplementary Table 16).

## Results

### Intestinal organoids derived from broiler and layer chicken embryos

**Fig. 1:**
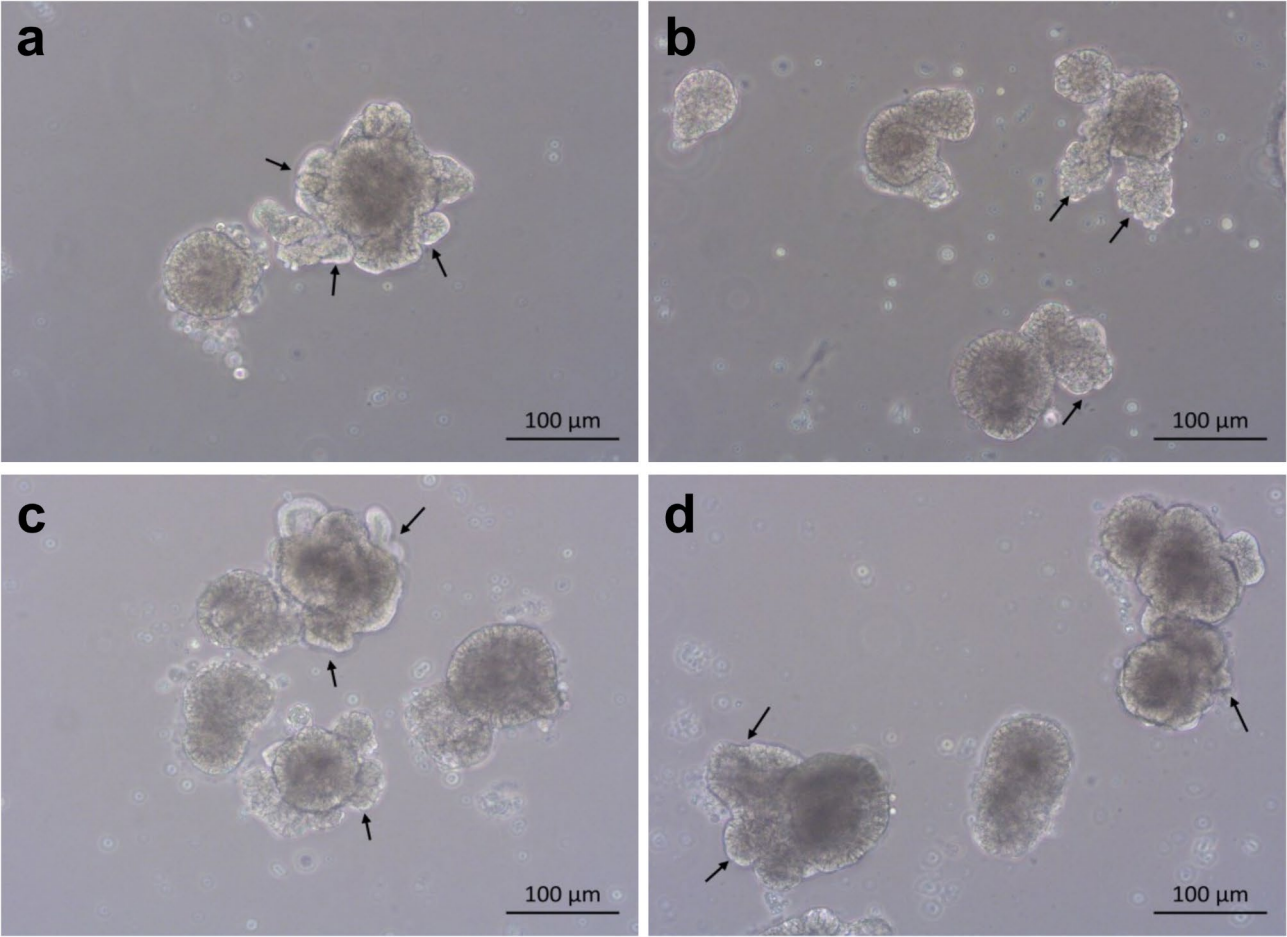
Development of multi-lobulated chicken organoids. Brightfield images of representative organoid cultures derived from ED19 layer and broiler embryos. Both layer (**a**, **b**) and broiler (**c**, **d**) organoids increased in size and exhibited multiple budding events at D3. Scale bar: 100 μm.

To advance understanding of the cellular composition of chicken 3D organoids containing an epithelial cell layer and lamina propria^7^, we performed scRNA-Seq on organoids derived from small intestine of ED19 layer and broiler embryos at day of isolation (D0) and day 3 (D3) of culture. Over the 3 day culture period, both broiler and layer organoids developed into large, multi-lobulated structures, each exhibiting multiple villus-like budding structures (Fig. 1).

We optimised our single cell isolation protocol through enzymatic digestion, using the Trypsin-EDTA solution recommended by 10X Genomics. The dissociated broiler and layer villi (D0) or organoids (D3) did not exhibit obvious differences in the percentage of single, live cells recovered (Supplementary Fig. 1). Optimisation of organoid dissociation and the subsequent analysis of cell viability via flow cytometry demonstrated ~ 15–20% of non-viable cells present in the cell preparations. To increase capture efficiency of live intact cells using 10X Chromium, live cells were isolated via FACS (see Methods). As reported below, our results demonstrate the capture of extensive cellular diversity and heterogeneity across the expected major cell lineages, indicating the effectiveness of our cellular isolation strategy.

### scRNA-Seq identifies five major cell lineages present in chicken 3D organoids

**Fig. 2:**
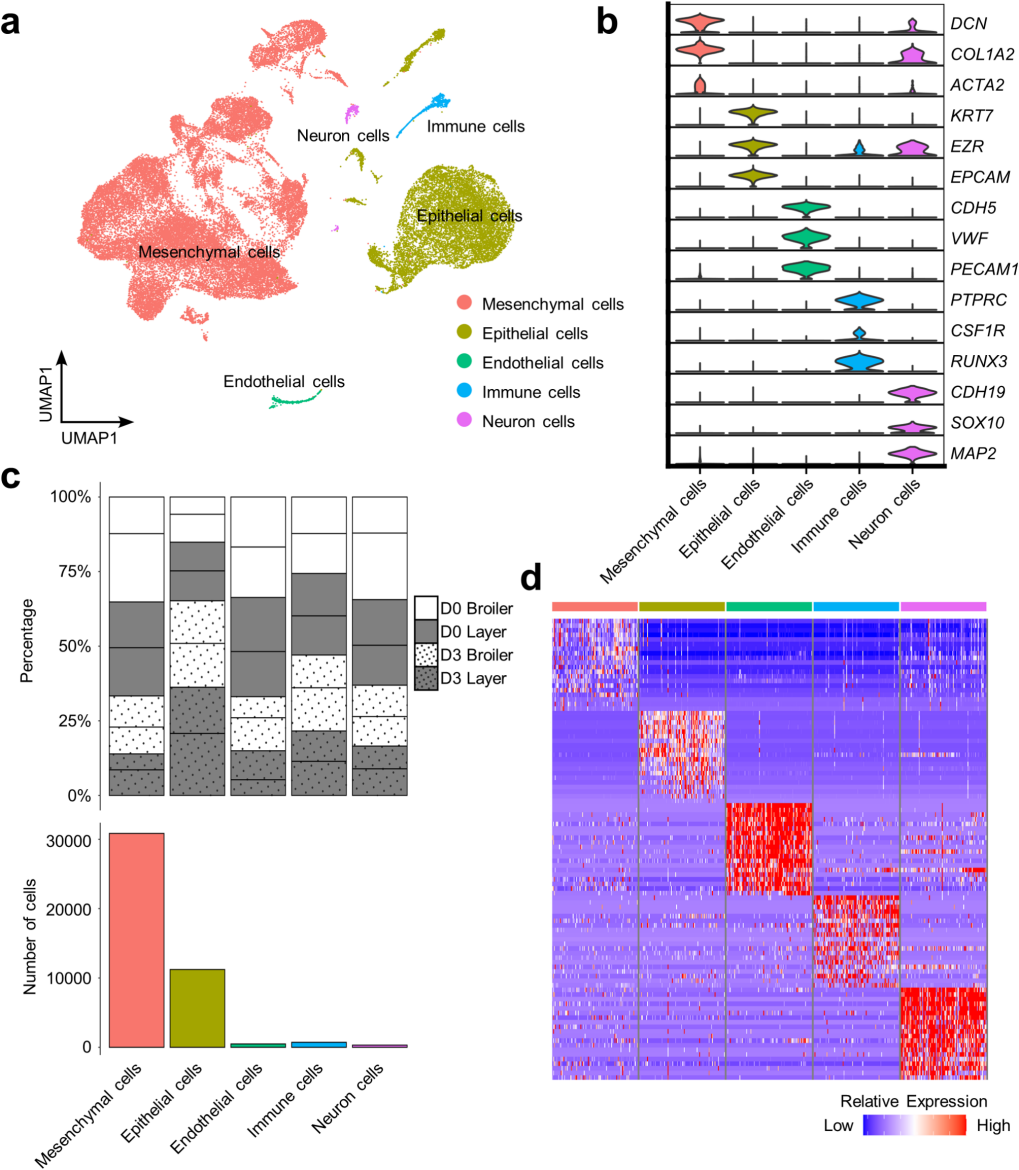
Major cell lineages identified in chicken intestinal organoids. (**a**) Clustering of 43,587 cells from D0 and D3 broiler (*n* = 4, 2 per timepoint) and layer (*n* = 4, 2 per timepoint) organoids. Cell lineages were inferred based on expression of marker genes. (**b**) Violin plots showing the expression level of canonical marker genes used to define the major cell lineages. (**c**) Top: Percentage contribution of each cell lineage for individual samples (key highlights organoid day and chicken breed and lines separate samples from different individuals within each category); Bottom: cell counts from each major lineage across all samples. (**d**) Heatmap of the top 20 DEGs for each major lineage. Columns represent cells; rows represent genes.

The organoids were divided into four groups for scRNA-Seq: D0 broiler (*n* = 2), D0 layer (*n* = 2), D3 broiler (*n* = 2) and D3 layer (*n* = 2). Following filtering and quality control, we captured the transcriptomes of 43,587 single cells across the samples, with an average 3,131 genes (s.d. 383 genes) per captured cell (Supplementary Table 1). Unbiased graph-based clustering^20^ identified five major cell lineages: mesenchymal, epithelial, immune, endothelial and neuronal cells, representing 71%, 26%, 2%, 1% and 1% of all cells, respectively (Fig. 2a; Supplementary Fig. 1–2).

Major cell lineages were assigned based on the expression of canonical mammalian and avian marker genes, supported by DGE tests across clusters (Fig. 2b–d; Supplementary Tables 2, 4). Mesenchymal and epithelial cells were the dominant lineages in each sample, although their distribution varied between breeds and timepoints (Fig. 2c; Supplementary Table 5). Broiler and layer organoids comprised on average 77% vs. 65% mesenchymal cells and 18% vs. 33% epithelial cells, respectively. D3 organoids exhibited a higher proportion of epithelial cells and a lower proportion of mesenchymal cells compared to D0 organoids (Fig. 2c). We also observed similar proportions of different cell types in each chicken line at the same time points (Fig. 2c).

Subsequent analyses specific to each major cell lineage revealed varying levels of heterogeneity. Hereafter, we focus on mesenchymal, epithelial and immune cells, which play key roles in nutrient absorption and pathogen defence. While multiple neuronal and endothelial subclusters were identified, each displayed few distinct marker genes, posing challenges in elucidating their biological significance (Supplementary Figs. 3, 4; Supplementary Tables 10, 11).

### Mesenchymal heterogeneity highlights the complexity of this lineage in chicken organoids

**Fig. 3:**
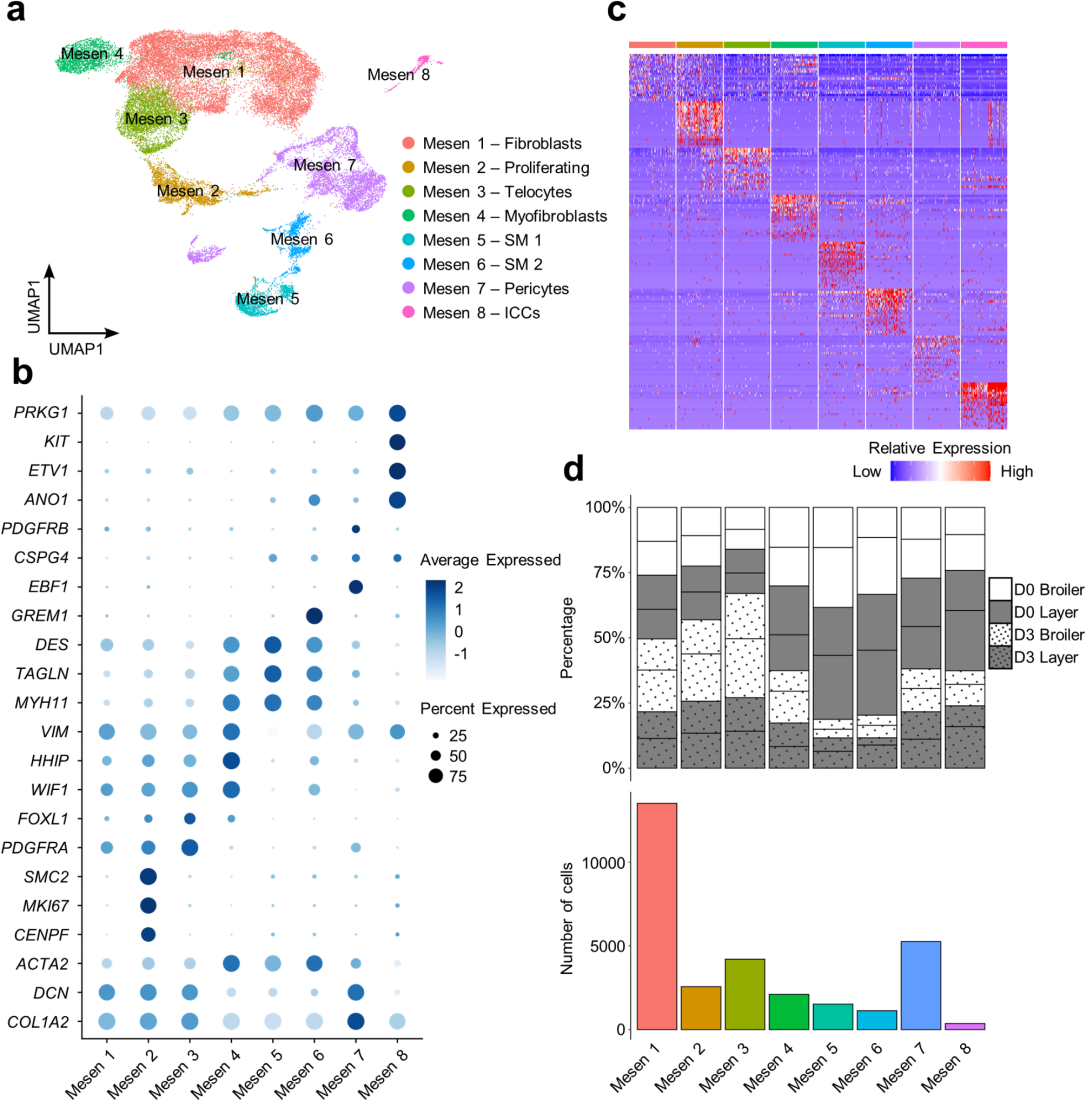
Heterogeneity in the mesenchymal cell lineage of chicken organoids. (**a**) Clustering of 30,661 mesenchymal cells (SM = smooth myocytes; ICCs = interstitial cells of Cajal). (**b**) Dotplot demonstrating expression of marker genes and notable cluster enriched genes. (**c**) Heatmap showing relative expression of the top 20 DEGs per cluster (rows) across the individual cells (columns). (**d**) Top: percentage of mesenchymal subpopulations for individual samples (lines separate samples from different individuals within each category shown on the key); bottom: cell counts for each mesenchymal subpopulation across all samples.

While chicken intestinal mesenchymal cells share similarities with their mammalian counterparts, their molecular and cellular properties are comparatively poorly defined^21^. We partitioned the chicken organoid mesenchymal cells into eight distinct populations (‘Mesen 1’ to ‘Mesen 8’), capturing candidate smooth myocytes (SM), pericytes, telocytes, myofibroblasts, interstitial cells of Cajal (ICCs), and potential mesenchymal stem cells (Fig. 3a), based on the expression of diverse niche factors and canonical mammalian markers (Fig. 3b,c; marker genes in Supplementary Table 7).

Mesen 1–3 were considered progenitor mesenchymal cells according to the expression of *PDGFRA*^22^, while *PDGFRA*^−^ Mesen 4 to 8 were annotated as mature myofibroblasts, SMs, pericytes and ICCs^22^. Mesen 1 (44% of mesenchymal cells) represent fibroblasts, based on the expression of genes encoding collagen (*COL1A2*)^23^ and decorin (*DCN*)^24^ (Fig. 3d; Supplementary Table 7). Mesen 2 (8% of mesenchymal cells) upregulated a range of proliferation-related genes (*CENPF*, *MKI67* and *SMC2*), suggesting they are proliferating (Fig. 3d; Supplementary Table 7). Mesen 3 (14% of mesenchymal cells) was annotated as WNT protein producing telocytes, with the highest *PDGFRA* expression and also expressed *FOXL1*^25^ and *WIF1*^26^. These cells were reported to be important for the maintenance of stem cells in the intestinal crypts^25^ (Fig. 3d; Supplementary Table 7).

Mesen 4–6 were annotated as myofibroblasts/SM according to the upregulation of myosin (*MYH11*) and actin (*ACTA2*) genes^27–30^. Mesen 4 (7% of mesenchymal cells) were annotated as myofibroblasts owing to high expression of genes encoding hedgehog interacting protein (*HHIP*), transgelin (*TAGLN*) and vimentin (*VIM*), along with a low level of *DES*, encoding the smooth myocytes marker desmin^31–34^ (Fig. 3d; Supplementary Table 7). *VIM*^low^*DES*^high^ Mesen 5 (5% of mesenchymal cells) and Mesen 6 (4% of mesenchymal cells) were considered separate SM populations, distinguished by uniquely expressing a gene encoding the BMP antagonist gremlin 1 (*GREM1*)^35^ (Fig. 3d; Supplementary Table 7). Mesen 7 (17% of mesenchymal cells) was identified as pericytes, characterized by the expression of *CSPG4*^36^, *EBF1*^37^ and *PDGFRB*^38^ (Fig. 3d; Supplementary Table 7). Mesen 8 (1% of mesenchymal cells) was annotated as ICCs based on the specific expression of *ANO1*^39,40^, *ETV1*^41^, *KIT*^42^ and *PRKG1*^43^ (Fig. 3d; Supplementary Table 7).

### Chicken organoids consist of six epithelial cell lineages

**Fig. 4:**
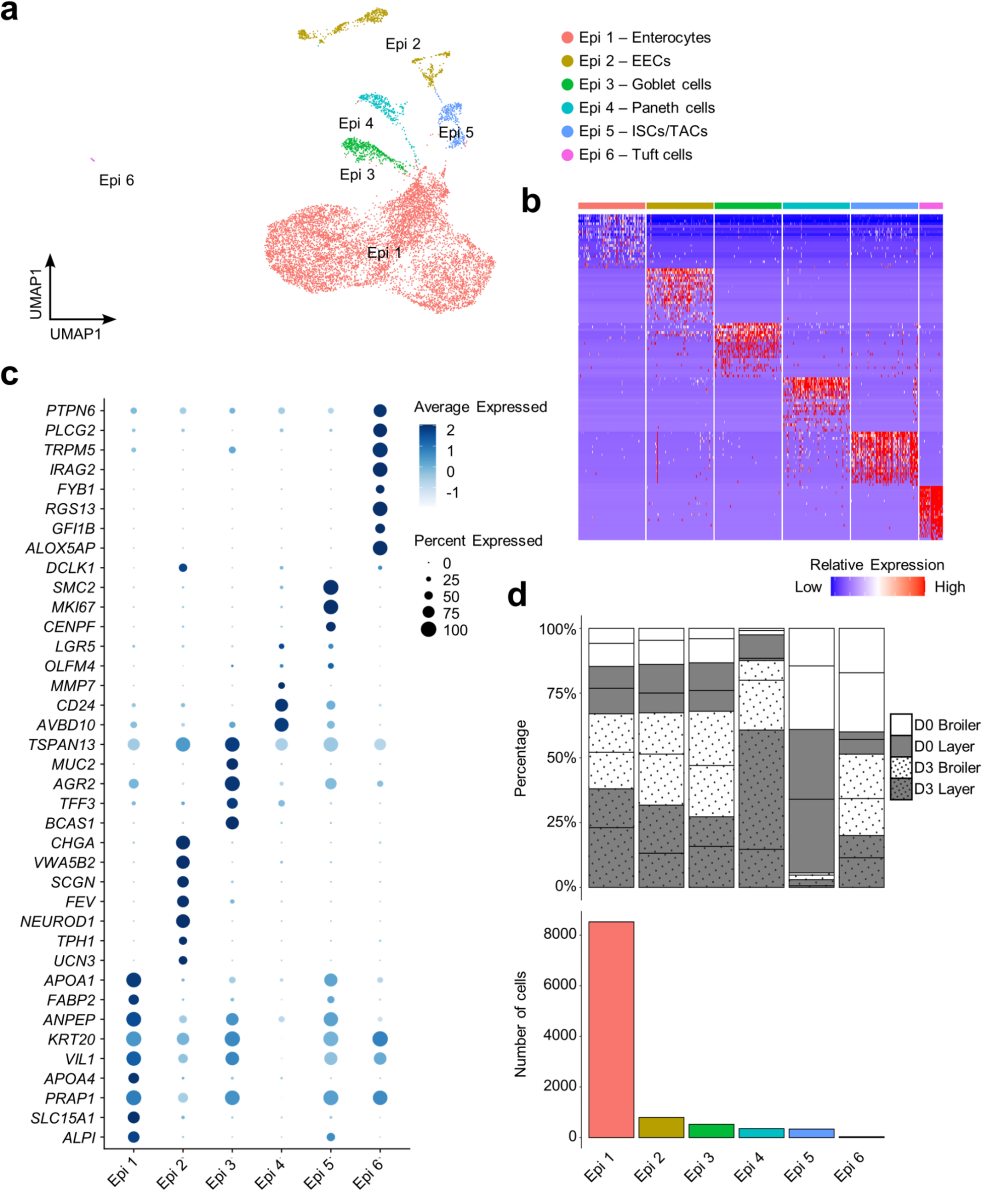
Heterogeneity in the epithelial cell lineage of chicken organoids. (**a**) Clustering of 10,576 epithelial cells (EECs = enteroendocrine cells; ISCs = intestinal stem cells; TACs = transit amplifying cells). (**b**) Heatmap shows the relative expression of top 20 DEGs per cluster (rows) across the individual cells (columns). (**c)** Dot plot demonstrating the expression of marker genes and notable cluster enriched genes. (**d**) Top: percentage of epithelial subpopulations for individual samples (lines separate samples from different individuals within each category shown on the key); bottom: cell counts for each epithelial subpopulation across all samples.

We annotated six epithelial subpopulations (“Epi 1” to “Epi 6”), including putative enterocytes, enteroendocrine cells (EECs), goblet cells, Paneth cells, tuft cells, transit amplifying cells (TACs), and intestinal stem cells (ISCs) (Fig. 4a,b; marker genes in Supplementary Table 8).

Epi 1, representing 81% of all epithelial cells, was annotated as enterocytes based on upregulation of chicken enterocyte marker genes *SLC15A1*^44^, *FABP2*^45^, *ALPI*, *PRAP1*, *APOA4*, *VIL1*, *KRT20*, *ANPEP* and *APOA1*^7^ (Fig. 4c,d; Supplementary Table 8). Epi 2 (8% of epithelial cells) was annotated as hormone-secreting EECs according to highly-specific expression of *UCN3*^46^, *TPH1*^47^, *NEUROD1*^48^, *FEV*^49^, *SCGN*^50^, *VWA5B2*^51^ and *CHGA*^52^ (Fig. 4c; Supplementary Table 8). Epi 3 (5% of epithelial cells) was annotated as goblet cells according to the expression of *BCAS1*^51^, *TFF3*^11,53^, *AGR2*^54^, *MUC2*^55^ and *TSPAN13*^56^ (Fig. 4c; Supplementary Table 8). Epi 4 (3% of epithelial cells) was annotated as Paneth cells by the specific expression of avian β-defensin (*AVBD10*)^57^ and classic avian Paneth-associated genes *CD24*^58,59^ and *MMP7*^7^ (Fig. 4c; Supplementary Table 8). Epi 5 (3% of epithelial cells) was annotated as ISCs/TACs by the expression of olfactomedin 4 (*OLFM4*)^44^, *LGR5* and proliferation related genes, including *CENPF*^60^, *MKI67* ^61^and *SMC2*^62^ (Fig. 4c; Supplementary Table 8). Epi 6 (0.3% of epithelial cells) was annotated as tuft cells according to the specific expression of characterized marker genes *DCLK1*^63^, *ALOX5AP*^64^, *GFI1B*^65^, *RGS13*^66^, *FYB1*^34^, *IRAG2*^67^, *TRPM5*, *PLCG2* and *PTPN6*^11^ (Fig. 4c; Supplementary Table 8).

### **Chicken 3D organoids consist of diverse enteroendocrine cell subpopulations**

**Fig. 5:**
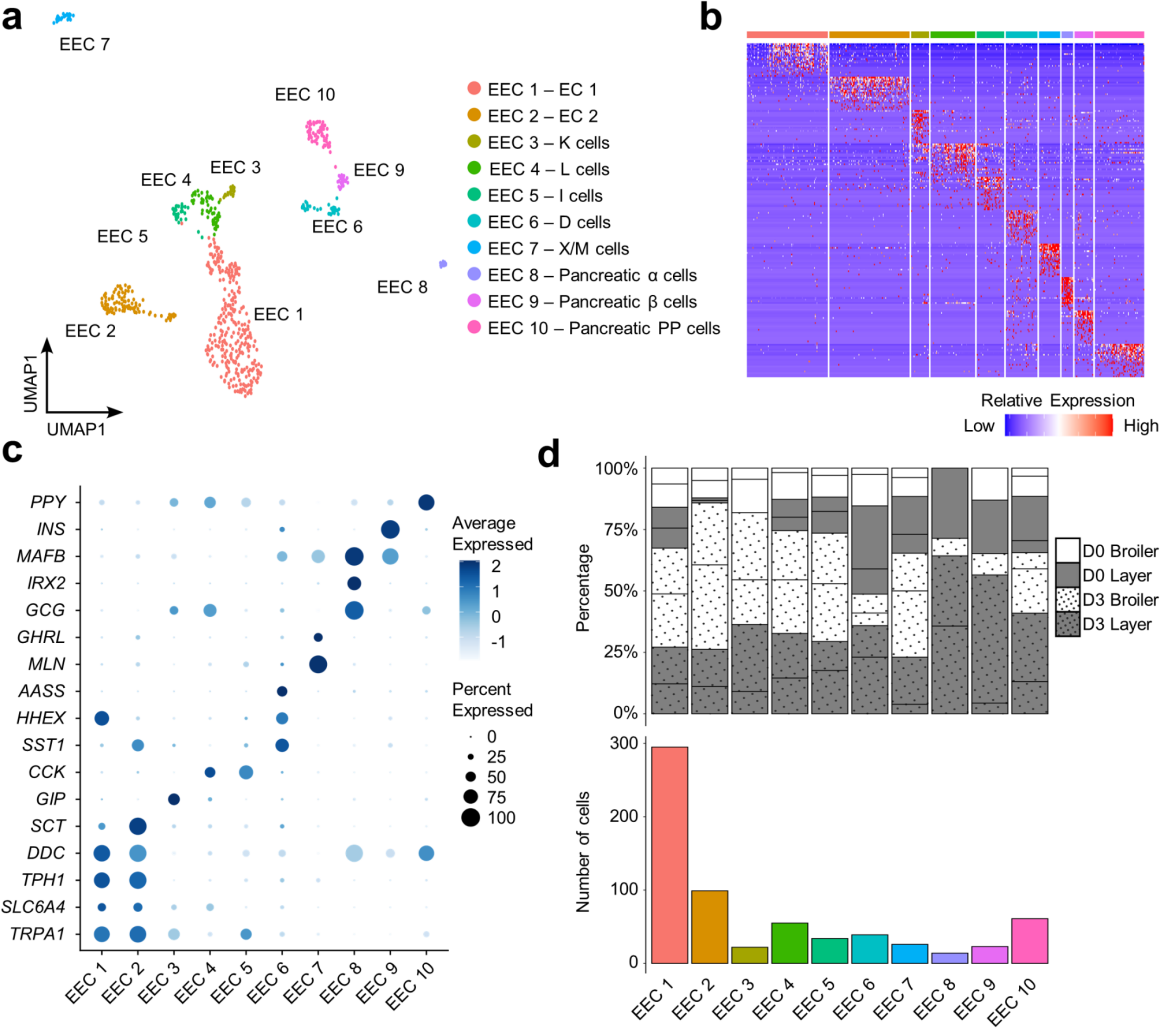
Heterogeneity in the enteroendocrine cell lineage of chicken organoids. (**a**) Clustering of 668 EECs (EC = enterochromaffin cells). (**b**) Heatmap shows the relative expression of top 20 DEGs per cluster (rows) across the individual cells (columns). (**c**) Dot plot demonstrating the expression of enteroendocrine cell subpopulation marker genes and notable cluster-enriched genes. (**d**) Top: percentage of enteroendocrine subpopulations for individual samples (lines separate samples from different individuals within each category shown on the key); bottom: cell counts for each enteroendocrine subpopulation across all samples.

To explore heterogeneity within the EEC cluster, we isolated and re-clustered Epi 2, which partitioned 668 EECs into 10 subclusters (“EEC 1” to “EEC 10”) (Fig. 5a–c; Supplementary Table 13). The relative proportion of cells comprising clusters EEC 1–7 was notably elevated in D3 compared to D0, and in broiler compared to layer organoids (Fig. 5d). Using canonical intestinal EEC markers, we annotated EEC 1–7 based on their expressed hormone products. EEC 1–2 were annotated as enterochromaffin cells, characterized by specific expression of the serotonin (5-HT) synthesis enzyme encoding genes *TPH1*^68^ and *DDC*^69^, together with the associated transcription factors *LMX1A*^70^, *SLC6A4*^71^ and *TRPA1*^72^ (Fig. 5c; Supplementary Table 13). EEC 2 (15% of EECs) showed up-regulation of the gene encoding secretin (*SCT*), suggesting further heterogeneity within the enterochromaffin cells^73^ (Fig. 5c; Supplementary Table 13). EEC 3 (3% of EECs) was annotated as K cells, based on specific expression of the gene encoding gastric inhibitory protein (*GIP*) (Fig. 5c; Supplementary Table 13), which stimulates insulin secretion^74^. EEC 4 (8% of EECs) was annotated as L cells, marked by the specific expression of the glucagon coding gene *GCG*^75^ (Fig. 5c; Supplementary Table 13). EEC 5 (5% of EECs) was annotated as appetite regulating cholestocystokinin (*CCK*) producing I cells^76,77^, based on specific expression of this key marker gene (Fig. 5c; Supplementary Table 13). EEC 6 (6% of EECs) was classified as somatostatin (*SST1*)^49^ expressing Delta (D) cell population, also expressing the canonical D cell marker *HHEX*^78^ (Fig. 5c; Supplementary Table 13). EEC 7 (4% of EECs) was annotated as X cells and enteroendocrine M cells based on specific expression of genes encoding motilin (*MLN*), which controls gut contractions in the inter-digestive state, and appetite-inducing ghrelin (*GHRL*)^79^ (Fig. 5c; Supplementary Table 13).

EEC 8 (2% of EECs) was annotated as pancreatic alpha cells according to the specific expression of *GCG*, *IRX2* and *MAFB*^80,81^ (Fig. 5c; Supplementary Table 13). EEC 9 (3% of EECs) was annotated as pancreatic beta cells according to the specific expression of *INS*^82^ (Fig. 5c; Supplementary Table 13), encoding insulin. EEC 10 (9% of EECs) was annotated as pancreatic polypeptide cells (PP) by the expression of the pancreatic polypeptide coding gene *PPY*^83^ (Fig. 5c; Supplementary Table 13).

### Immune cell heterogeneity in the chicken organoids

**Fig. 6:**
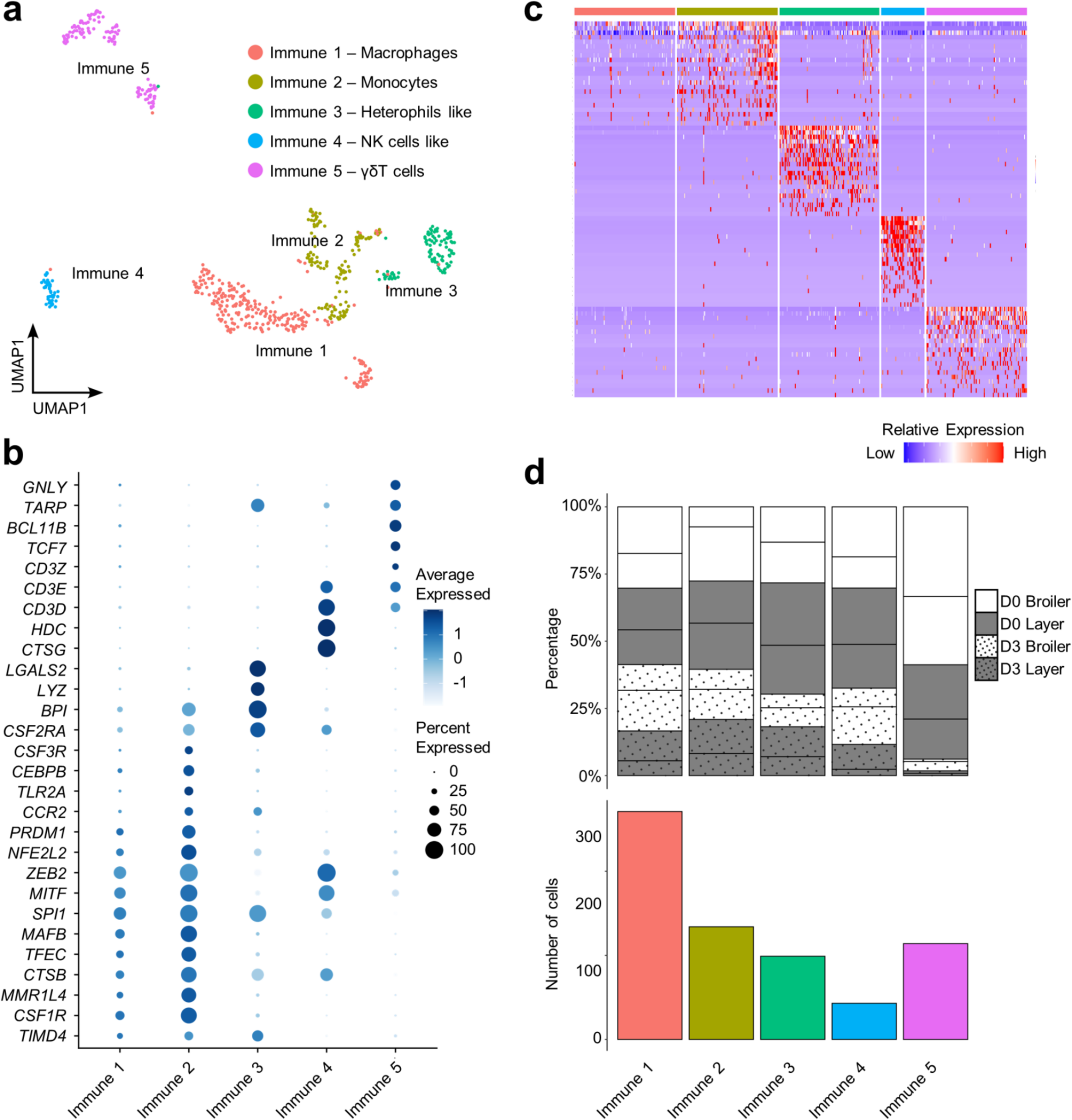
Heterogeneity in the immune cell lineage of chicken organoids. (**a**) Clustering of 661 immune cells (NK cells = Natural Killer cells). (**b**) Scaled gene expression of canonical marker genes across all immune cells. (**c**) Heatmap showing relative expression of the top 20 DEGs per cluster (rows) across the individual cells (columns). (**d**) Top: percentage of immune subpopulations for individual samples (lines separate samples from different individuals within each category shown on the key); bottom: cell counts for each immune subpopulation across all samples.

Previous work has identified the presences myeloid and lymphoid cells in chicken organoids through immunostaining using relevant markers^7^. Our analysis supports the presence of five distinct immune clusters (“Immune 1” to “Immune 5”) (Fig. 6a; marker genes in Supplementary Table 9) with identities assigned using established myeloid and lymphoid marker genes (Fig. 6b) and supported by examining marker genes defined by DGE tests across clusters (Fig. 6c). Each sample type showed different contributions to the immune subpopulations, particularly notably comparing D0 and D3 (Fig. 6d; Supplementary Table 9).

Immune 1 to 3 were annotated as myeloid cells, according to specific expression of *TIMD4*^84^ and the colony stimulating factor encoding genes *CSF1R*, *CSF2R*, *CSF3R*^85^ (Fig. 6b; Supplementary Table 9). Specific expression of *MMR1L4* (*MRC1L-B*), encoding a member of the chicken mannose receptor family was used to assign Immune 1 and 2 as monocytes/macrophages^86,87^. Immune 1 (41% of immune cells) was annotated as macrophages according to the expression of *CSF1R*^88^, *CTSB*^89^, *TFEC*^90^, *MAFB*^91^, *SPI1*^92^, *MITF*^93^ and *ZEB2*^94^. Immune 2 (20% of immune cells) was annotated as monocytes owing to the expression of *CSF3R*, *CCR2*, *TLR2A*, *MMR1L4*, and transcription factor encoding genes *CEBPB*, *NFE2L2*, *PRDM1* and *TFEC*, as reported elsewhere^84^ (Fig. 6b; Supplementary Table 9). Immune 3 (15% of immune cells) was considered as putative granulocytes, functionally equivalent to mammalian neutrophils, owing to the low expression of *CSF1R*^95^ and high expression of *CSF2R*^96^, alongside genes encoding bactericidal permeability-increasing protein (*BPI*)^97^, lysozyme (*LYZ*)^98^ and galectin 2 (*LGALS2*)^99^ (Fig. 6b; Supplementary Table 9). The identity of Immune 4 (8.45% of immune cells) was not conclusively established.

Among the putative lymphoid cells, Immune 4 (7% of immune cells) and Immune 5 (17% of immune cells) were identified as NK cells (Natural Killer cells) and T cells, respectively, with no detection of B cells (Fig. 6b; Supplementary Fig. 5). Distinguishing T cells and NK cells was challenged by the overlapping expression of *CD3*, *IL2RA* and *LAMP1*^100,101^. Immune 4 was identified as putative NK cells, according to the expression of *CD3D*, *CD3E*, *CTSG*^102^ and the absence of *CD3Z*, *TCF7* and *BCL11B* (Fig. 6b; Supplementary Table 9). Immune 5 (16% of immune cells) was annotated as γδT cells, supported by the expression of all three CD3 complex coding genes (*CD3D*, *CD3E* and *CD3Z*)^7^, *TCF7*^103^, *BCL11B*^104^, *GATA3*^86^, *MAF*^86^, TCR γ chain (*TARP*)^105^ and the gene encoding granulysin (*GNLY*) (Fig. 6b; Supplementary Table 9).

### Transcriptomic differences between broiler and layer organoids

**Fig. 7:**
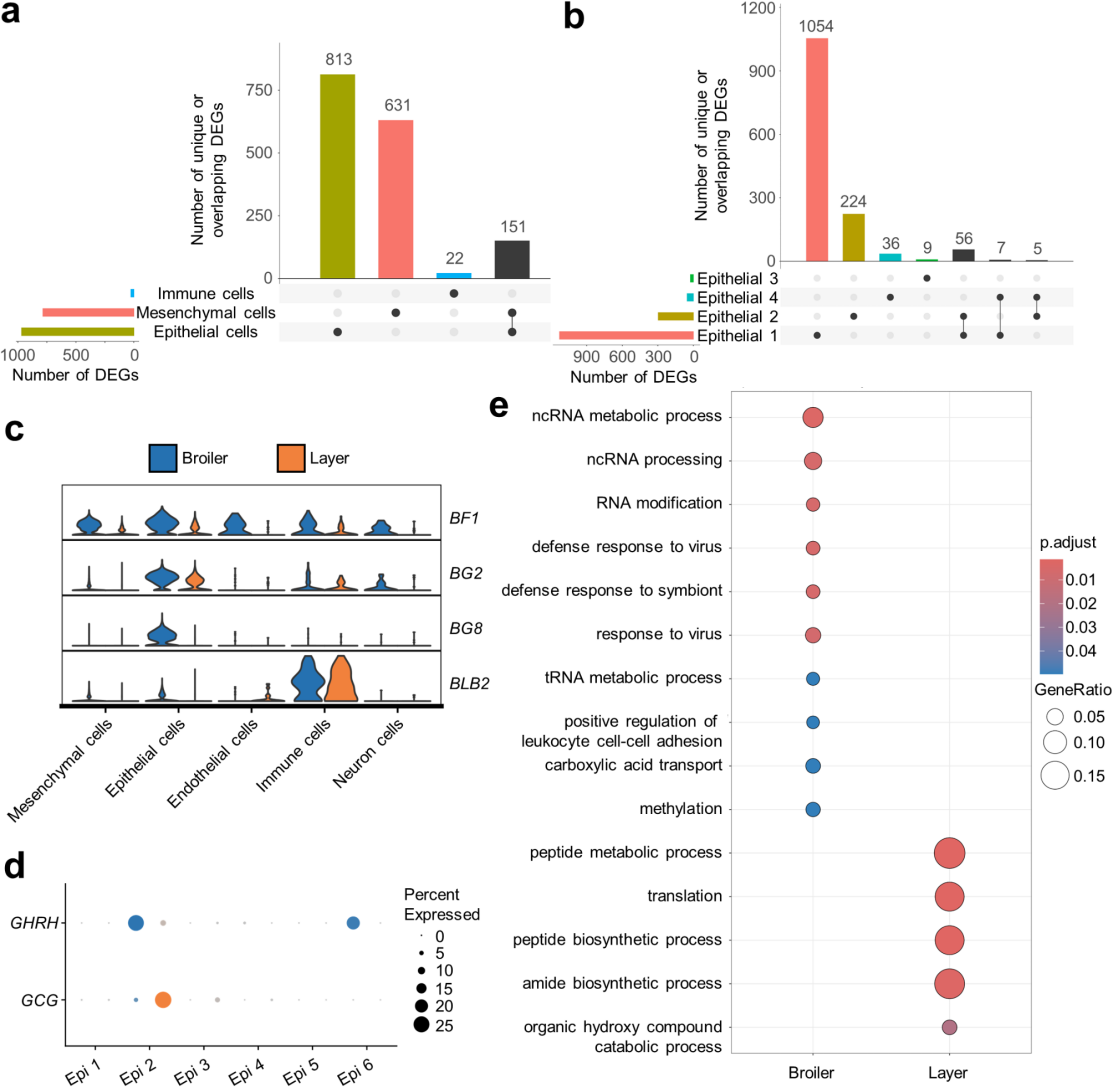
Transcriptomic comparison of broiler and layer D3 organoids. Upset plots show overlap between DEGs identified separately in the major organoid cell lineages (**a**) or epithelial subpopulations (**b**). Also shown (**c**) is relative gene expression of MHC associated genes in each major cell lineage. (**d**) Expression of two appetite regulation hormone encoding genes in epithelial cell subpopulations. (**e**) Top ten enriched GO terms among the upregulated genes in broiler and layer enterocytes (Epi 1).

Given the long-term selection for distinct broiler and layer phenotypes, which has impacted feed intake and nutrient utilization^106^, as well as immune responses^107^, we asked if intestinal organoids showed transcriptomic differences between the two breeds, and if so, in which cell types. We firstly performed differential expression tests for each of the five major cell lineages, revealing the strongest transcriptome changes in epithelial cells (968 DEGs), followed by mesenchymal (782 DEGs) and immune cells (26 DEGs) (Fig. 7a; Supplementary Table 14). These differences were predominantly cell-type specific, with 84%, 81%, and 88% of the DEGs being restricted to the epithelial, mesenchymal and immune lineages, respectively (Fig. 7a). Most of the remaining DEGs were common to epithelial and mesenchymal cells, around 16% and 19%, respectively (Fig. 7a). The endothelial and neuronal cell populations showed only 3 DEGs each (Supplementary Table 14). Given the known differences in immunity between broiler and layers^107^, alongside the importance of the MHC system for differences in immune response and pathogen resistance among chicken breeds^108^, it was notable that broiler organoids showed strong upregulation of MHC associated genes *BF1* (class I heavy chain), *BLB2* (class II beta chain), *BG2* and *BG8*^109^, especially within epithelial cells, but also across multiple cell types (Fig. 7b).

Given the strongest transcriptomic difference in the epithelial lineage, we further resolve transcriptomic differences across the distinct epithelial subpopulations. We identified 1,129, 296, 24, and 52 DEGs in Epi 1 (enterocytes), 2 (EECs), 3 (goblet cells) and 4 (Paneth cells) (no DEGs in Epi 5 and 6) (Fig. 7c; Supplementary Table 15). As observed in the major cell lineage analysis, the majority of epithelial DEGs were cell type-specific, most strikingly with > 93% of enterocyte (Epi 1) DEGs restricted to this subpopulation (Fig. 7c). Interestingly, in the EECs, we identified significant upregulation of *GHRH* (encoding growth-hormone-releasing hormone) in broilers, whereas layer organoids upregulated *GCG* (Fig. 7d).

Finally, GO enrichment tests were performed for each epithelial subpopulation to explore biological processes overrepresented among genes showing upregulation in either broiler or layer organoids (Fig. 7e; Supplementary Table 16). The largest number of significant GO terms was identified in enterocytes (Epi 1), indicating enrichment of broiler-upregulated genes with roles in viral defence, non-coding RNA regulation, methylation and immune cell adhesion (Fig. 7e; Supplementary Table 16). Layer-upregulated enterocyte genes were enriched in GO terms associated with translation and peptide metabolism/biosynthesis (Fig. 7e; Supplementary Table 16). A limited number of GO terms were enriched in other epithelial subpopulations, including biological processes linked to cell projection for layer-upregulated genes in EECs (Epi 2), cell size regulation for layer-upregulated genes in goblet cells (Epi 3) and smooth muscle cell migration for broiler-upregulated genes in Paneth cells (Epi 4) (Supplementary Table 16).

## Discussion

Although immunohistochemistry and bulk RNA-Seq analysis have highlighted the conserved and divergent features of major cell lineages in chicken organoids, previous investigations lack the high-resolution provided by scRNA-Seq. Here, we present a comprehensive cell atlas of chicken intestinal organoids, comprising 43,587 cells from eight morphologically similar chicken organoids collected from D0 to D3. The identification of five major cell lineages shows a strong correlation to results from past bulk transcriptomics and histology analysis^6^.

The intricate process of intestinal development and the fine-tuned balance between proliferation and differentiation within the mature organ are understood to emerge from dynamic interactions between epithelial cells - localized within the surface epithelium - and mesenchymal cells, which span from the subepithelial lamina propria to the serosa. The maintenance, functionality, and proliferation of crypt *LGR5*^+^ ISCs and TACs relies on diverse niche factors, including canonical WNT ligands^110,111^, R-spondins (RSPOs)^112^, and bone morphogenetic proteins (BMPs)^113,114^. Mammalian sub-epithelial mesenchymal cells are primary sources of these pivotal signals, which regulate mitosis inhibition and prompt terminal differentiation of epithelial cells^115–117^. In chicken organoids with a basal out orientation, the addition of RSPO1 and WNT3 was demonstrated to sustain organoid longevity^118^, however in our organoids (with an apical out orientation) these pivotal signals originate from the subepithelial mesenchymal cells present in the culture. In this study, we classified mesenchymal cells into eight subpopulations encompassing all components of the lamina propria mesenchyme, including fibroblasts, telocytes, myofibroblasts, pericytes, smooth myocytes, and ICCs. Thus, our chicken organoid model recaptures a complex subepithelial cell layer structure or lamina propria, closely mirroring in vivo anatomical organization^119^.

In mammals, despite the variability among epithelial cells across different regions of the small intestine, those derived from crypt *LGR5*^+^ ISCs can generally be categorized into two functional groups: absorptive (i.e. enterocytes and microfold cells) and secretory (i.e. enteroendocrine, goblet, Paneth and tuft cells). These epithelial subpopulations have been identified in chickens, where they play comparable roles in digestion, nutrient absorption, and initiating immune responses to pathogens^7,120^. All the expected epithelial subpopulations were identified in our organoids by scRNA-Seq using known chicken marker genes. While microfold cells have been reported in adult chicken intestines, no microfold cell clusters were identified using the known chicken markers *ANXA10*, *CD44*, *SPI1* and *SOX8*^120^ (Supplementary Fig. 6; Supplementary Table 2), which aligns with histology^7^. Although a distinct undifferentiated epithelial population could not be identified solely with the commonly used ISC/TAC marker *LGR5*, a small proportion of cells within the putative ISC/TAC subpopulation (Epi 5), expressed both *LGR5* and *OLFM4*, consistent with in situ hybridization results^44^.

EECs secrete hormones that signal to the local enteric nervous system and to several organs including pancreas and brain, regulating fundamental processes including food intake, insulin secretion, digestive enzyme release, and bowel motility^11^. In chickens, EECs are known to exhibit significant heterogeneity, akin to their mammalian counterparts, albeit with distinct physiological functions attributed to their hormone signalling pathways^121–123^. Our comprehensive analysis identified all expected intestinal EEC types, including ECs, K cells, L cells, I cells, D cells, and X/M cells. A chicken subpopulation analogous to mammalian N cells was not identified, with the signature mammalian N cell marker gene *NTS*^124^ (encoding neurotensin) expressed in almost all EEC subpopulations (Supplementary Table 13). Notably, our analysis also revealed candidate pancreatic EEC subpopulations (EEC 8–10). Like their mammalian counterparts, most avian pancreatic EECs are composed of glucagon-producing alpha cells, insulin-producing beta cells, somatostatin-producing D cells, and polypeptide-producing PP cells^125–128^. This observation of pancreatic endocrine cells could be due to contamination from nearby islets of Langerhans during the duodenum sampling process. However, the absence of other pancreatic cells (acinar cells and duct cells) does not exclude other possibilities. Mammalian intestinal EECs can produce pancreatic polypeptide locally^129,130^, while human intestinal EECs can produce insulin during fetal development^130^. However, such processes have not been reported in avian literature. The potential transition of intestinal endocrine cells into pancreatic endocrine cells is also plausible, as ectopic expression of *PAX4* in mouse intestinal organoid D cells facilitates a reprogramming into insulin-secreting pancreatic beta-like cells^131^. These findings underscore the importance of exercising caution when extrapolating knowledge from organoid-like structures to in vivo settings, as they may contain unexpected cell types and hence cellular interactions.

Among the immune cells identified in this study, macrophages and monocytes were straightforward to define using gene markers. Annotating potential granulocytes, and NK/T cells posed greater challenges. It has previously been shown that several lysosomes and cathelicidin encoding genes are enriched in chicken granulocytes but not monocytes^98^; however, only *LYZ* showed elevated expression in subpopulation Immune 3. Together with the expression of mammalian neutrophil key regulators *BPI* and *LGALS2*, we identified characteristics of granulocytes within this population. Dendritic cells or B cells were not identified among the immune cells analysed (Supplementary Fig. 5; Supplementary Table 2). It’s unsurprising that B cells are lacking, due to the large emigration of B cells from the bursa of Fabricius beginning around hatching^132,133^. The dominance of γδ T cells in chicken organoids was expected, due to their migration from the thymus to the intestinal epithelium by ED15, whereas αβ T cells only begin to migrate from ED19^134^.

Beyond the well-known differences in economically significant production traits between broilers and layers, layer chickens appear to have a more robust immune system, as evidenced by their longer lifespan and enhanced immune function in adulthood^107^. To investigate whether these traits are mirrored in the composition and function of organoid cells, we assessed the morphology and sequenced organoids derived from both chicken breeds. No obvious differences in morphology or growth rate were observed among D3 organoids, while in vivo broiler embryos have a larger villus areas compared to layer embryos^135^. Broiler organoids displayed increased expression of MHC associated genes *BF1*, *BLB2*, *BG2* and *BG8* within epithelial cells. Although considerable speculation exists regarding the immunological roles of chicken BG genes^136^—owing to their similarity to ancestral butyrophilin genes—their expression indicate specific cell and tissue functions^109^. Combined with the role of MHC genes in epithelial renewal observed in mammals^137^, our findings may reflect accelerated epithelial development in broilers rather, than an amplified immune response. In terms of hormonal regulation within enteroendocrine cells, broilers exhibited upregulation of the orexigenic neuropeptide *GHRH*, while layers upregulated the anorexigenic neuropeptide *GCG*, consistent with the broiler’s stronger appetite and higher food intake. Other satiety influencing genes expressed in post-hatch chicken intestines, such as *CCK* and *PYY* (ENSGALG00015025746)^138^, showed no significant differences between breeds (Supplementary Table 15). Our GO enrichment results also point to major functional differences in epithelial cell between broilers and layers, particularly in enterocytes, where genes upregulated in broilers included immune genes.

In conclusion, this study provides the first detailed single cell transcriptomic atlas of chicken intestinal organoids, highlighting extensive cellular diversity and potential differences between broilers and layers, along with thousands of marker genes for different intestinal cell types. Our findings provide novel insights into chicken intestinal biology and breed differences, and in the longer-term will support the development of more precise poultry breeding and health management strategies derived from research applications leveraging intestinal organoid models.

## Electronic supplementary material

Below is the link to the electronic supplementary material.

Supplementary Material 1

Supplementary Material 2

## Data Availability

All data generated in this study is available in the GEO database (https://www.ncbi.nlm.nih.gov/geo/) with the GEO accession: GSE283090. All data required to interpret and use the findings of this study is provided within the manuscript or its supplementary files. Requests for additional data access should be directed to the corresponding author Daniel J. Macqueen.

## Code availability

Code for analyses reported available at GitHub - JianxuanS/Chicken_organoids.
