# Supplementary material for: Cellular landscape of avian intestinal organoids revealed by single cell transcriptomics
Source: Sci Rep. 2025 Apr 2;15:11362. doi: 10.1038/s41598-025-95721-4 (PMC11965369; doi:10.1038/s41598-025-95721-4)
Supplement: Supplementary file 1 — Supplementary Material 1 [file 41598_2025_95721_MOESM1_ESM.docx]

Supplementary Information

**Cellular landscape of avian intestinal organoids revealed by single cell transcriptomics**

Jianxuan Sun, Dominika Borowska, James J. Furniss, Kate Sutton, Daniel J. Macqueen, Lonneke Vervelde

*This document includes:*

**Supplementary Figure 1**. Sorting of single-cell suspensions from digested villi and organoids stained with Sytox Blue live/dead stain

**Supplementary Figure 2**. Expression of canonical marker genes used to define the five major cell lineages

**Supplementary Figure 3**. Heterogeneity in the endothelial cell lineage of chicken organoids

**Supplementary Figure 4**. Heterogeneity in the neuronal cell lineage of chicken organoids

**Supplementary Figure 5**. Expression of canonical chicken B cell markers in immune cell subpopulations

**Supplementary Figure 6**. Expression of canonical chicken bursa microfold cell markers in epithelial cell subpopulations

*Included as a seperate file:*

**Supplementary Table 1.** Statistics on scRNA-seq data and mapping outcomes for each organoid sample

**Supplementary Table 2.** Marker genes defined a priori and used for supervised clustering of the 5 major organoid cell lineages and potential subpopulations. We include a description concerning the source of evidence defining each marker gene. Highlighted subpopulations (yellow labelled) were not identified in this study.

**Supplementary Table 3.** Broiler orthologues of layer coding genes based on Ensembl rapid release

**Supplementary Table 4.** Marker genes defined by differential expression tests for 5 major cell lineages within the chicken organoids. Averaged gene expression levels for each cell lineage are provided in a heatmap format. The percentage of cells where the gene is detected within or outside each cell lineage is represented as pct.1 and pct.2, respectively

**Supplementary Table 5.** Summary statistics for scRNA-seq data generated for eight chicken organoid samples

**Supplementary Table 6.** Marker genes defined *a priori* and used for supervised clustering of cell sub-populations defined in EEC specific clustering analyses. We include a description concerning the source of evidence defining each marker gene

**Supplementary Table 7.** Marker genes defined by differential expression tests for 8 Mesenchymal cell clusters

**Supplementary Table 8.** Marker genes defined by differential expression tests for 6 Epithelial cell clusters

**Supplementary Table 9.** Marker genes defined by differential expression tests for 5 Immune cell clusters

**Supplementary Table 10.** Marker genes defined by differential expression tests for 6 Endothelial cell clusters

**Supplementary Table 11.** Marker genes defined by differential expression tests for 5 neuronal cell clusters

**Supplementary Table 12.** Summary statistics of cell sub-populations of five major cell lineages and enteroendocrine subpopulations across the eight samples; including the number and proportion of cells per sub-population

**Supplementary Table 13.** Marker genes defined by differential expression tests for 10 EEC cell clusters

**Supplementary Table 14.** Differentially expressed genes for D3 broiler vs layer across the major cell lineages. Genes with log2FC values > 0 were considered up-regulated in the broiler samples, while those with log2FC values < 0 were considered up-regulated in the layer samples

**Supplementary Table 15.** Differentially expressed genes for D3 broiler vs layer across the epithelial sub-populations. Genes with log2FC values > 0 were considered up-regulated in the broiler samples, while those with log2FC values < 0 were considered up-regulated in the layer samples

**Supplementary Table 16.** GO enrichment test for each epithelial subpopulation restricted to Biological Function terms. GeneRatio represents the proportions of genes in the input lists associated with the given GO term. BgRatio represents the proportions of given GO term associated genes relative to the background gene set. geneID column shows genes contributing to each term


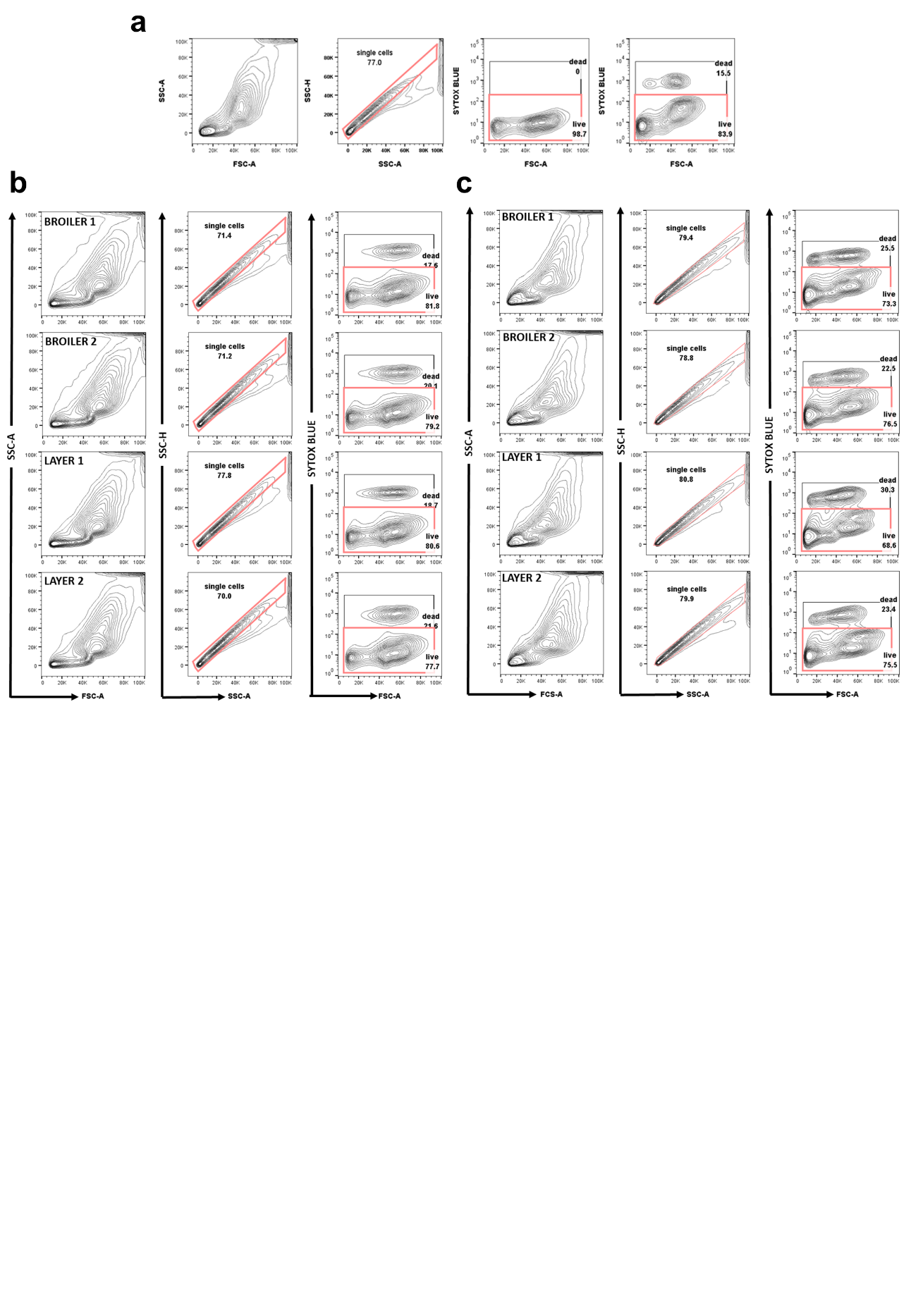


**Supplementary Figure 1**. **Sorting of single-cell suspensions from digested villi and organoids stained with Sytox Blue live/dead stain.** **a** Data were gated for singlets and live cells. The same gating strategy applies to all samples in the figure. **b** Percentage analysis of single and live cells obtained from two batches of villi derived from broiler and layer embryos. **c** Percentage analysis of single and live cells obtained from two batches of organoids derived from broiler and layer villi and cultured for 3 days.


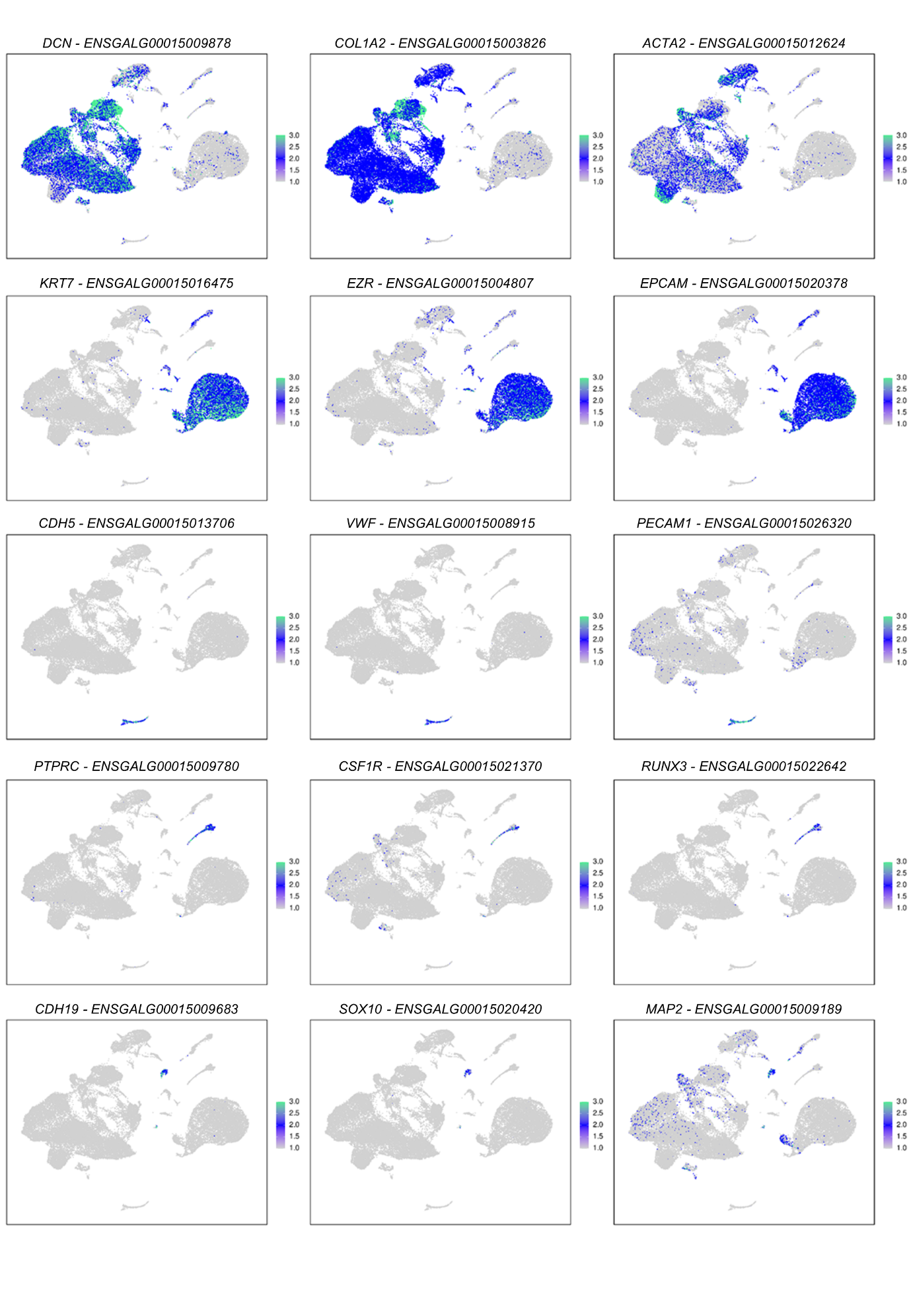


**Supplementary Figure 2**. **Expression of canonical marker genes used to define the five major cell lineages.**


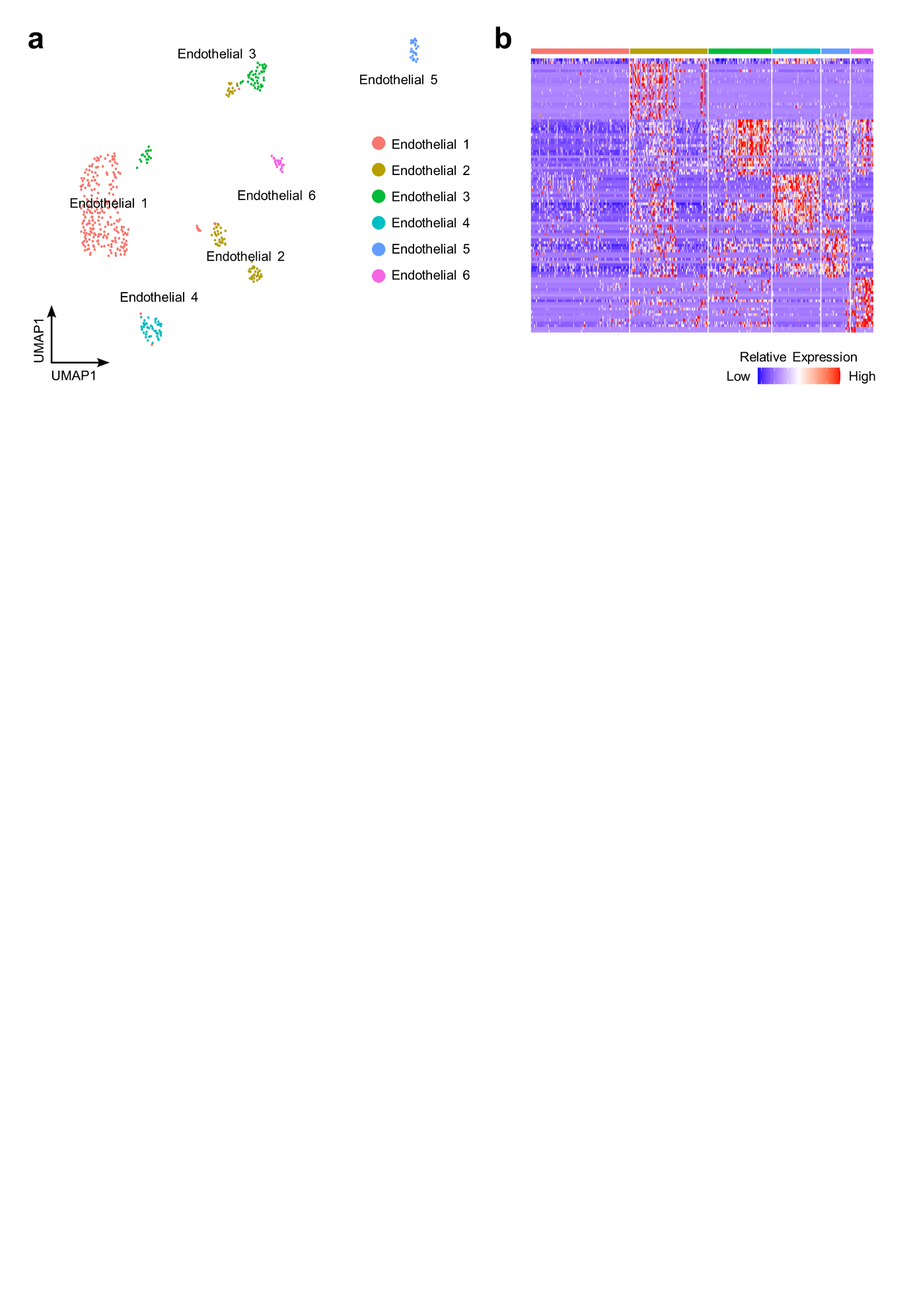


**Supplementary Figure 3**. **Heterogeneity in the endothelial cell lineage of chicken organoids**. **a** Clustering of 489 endothelial cells. **b** Heatmap shows the relative expression of top 20 DEGs per cluster (rows) across the individual cells (columns).


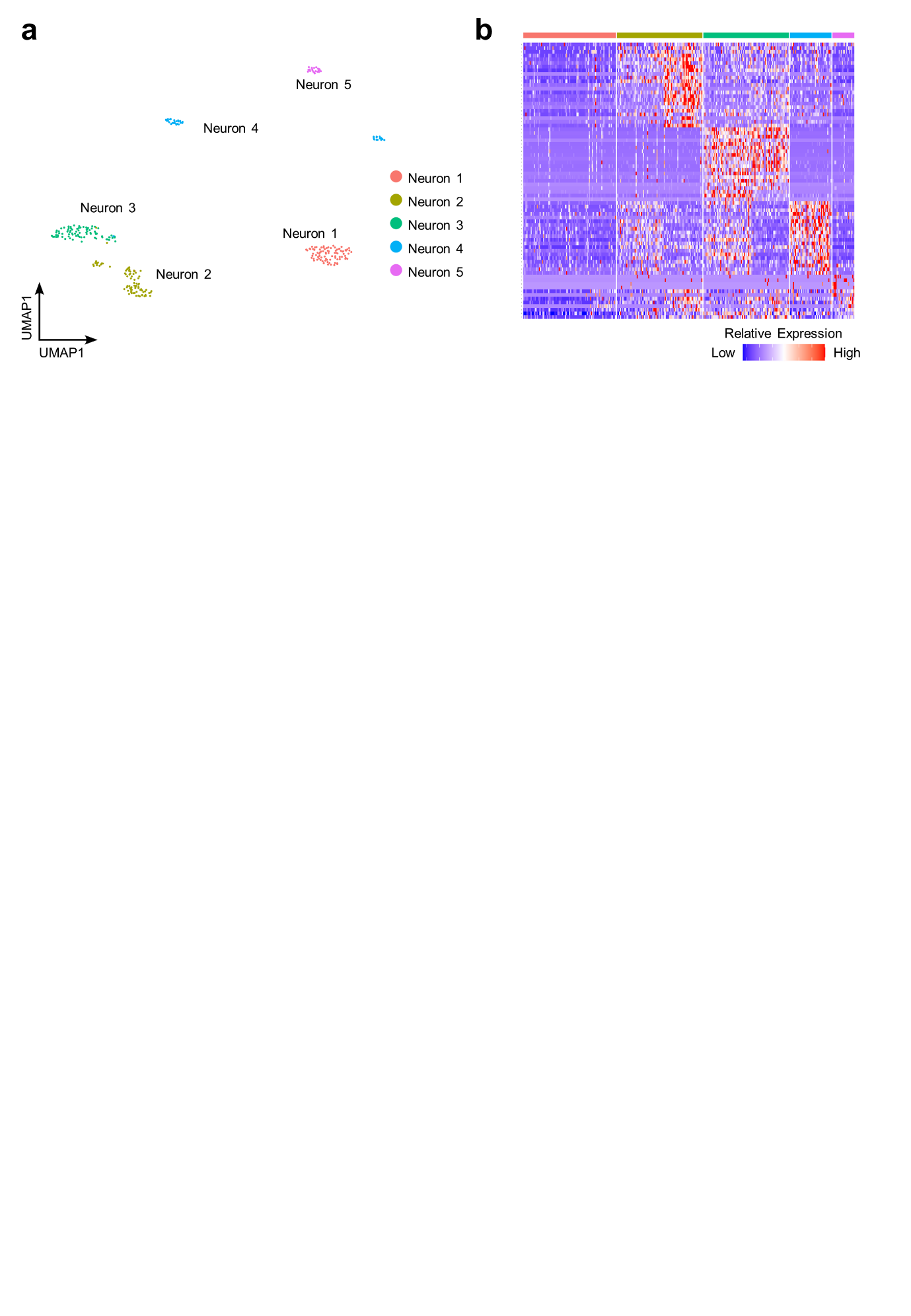


**Supplementary Figure 4**. **Heterogeneity in the neuronal cell lineage of chicken organoids.** **a** Clustering of 489 neuronal cells. **b** Heatmap shows the relative expression of top 20 DEGs per cluster (rows) across the individual cells (columns).

**
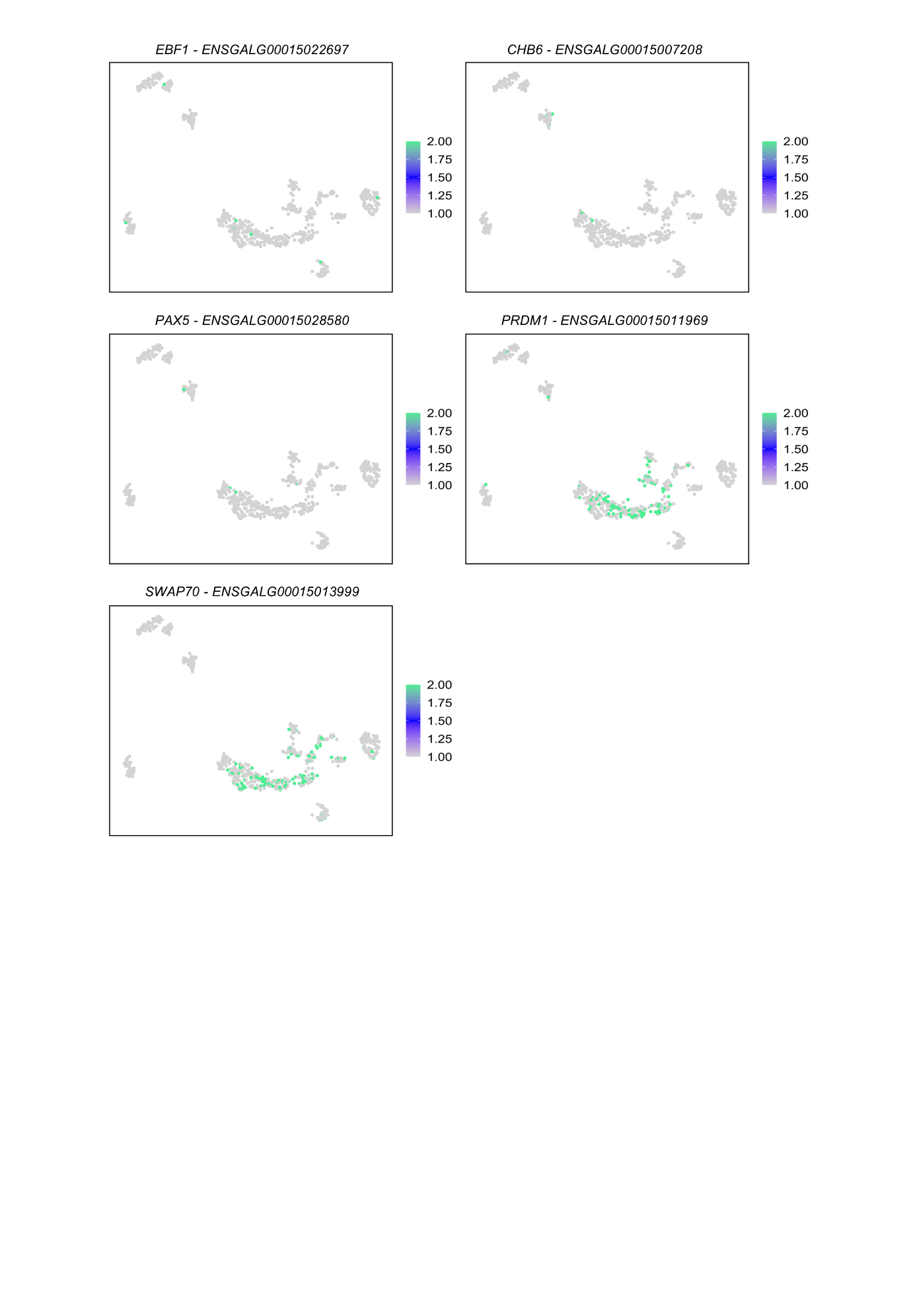
**

**Supplementary Figure 5. Expression of canonical chicken B cell markers in immune cell subpopulations.**


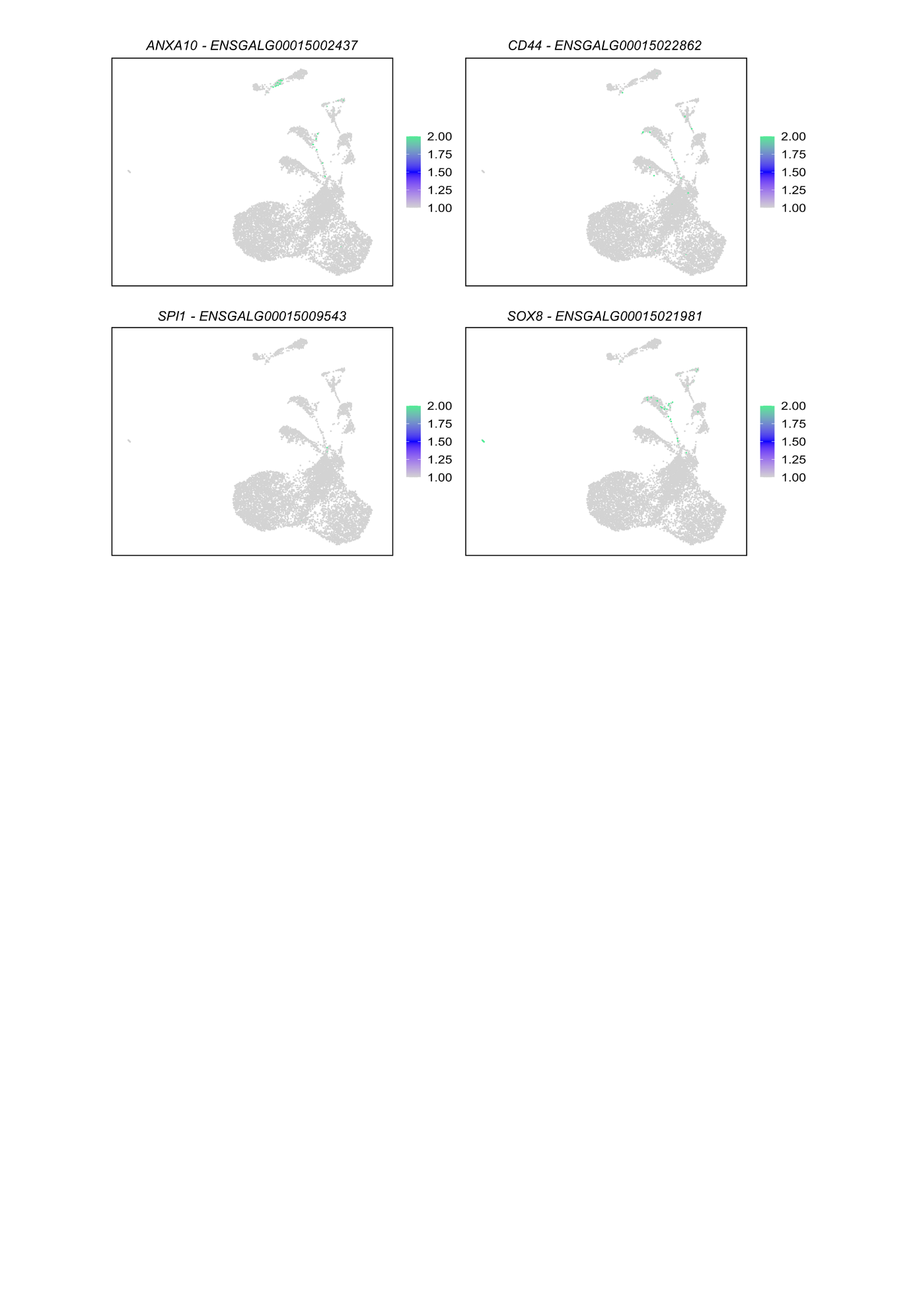


**Supplementary Figure 6**. Expression of canonical chicken bursa microfold cell markers in epithelial cell subpopulations.
